# Parallel evolution controlled by adaptation and covariation in ammonoid cephalopods

**DOI:** 10.1186/1471-2148-11-115

**Published:** 2011-04-29

**Authors:** Claude Monnet, Kenneth De Baets, Christian Klug

**Affiliations:** 1 Paläontologisches Institut und Museum, Universität Zürich, Karl Schmid Strasse 4, 8006 Zürich, Switzerland

## Abstract

**Background:**

A major goal in evolutionary biology is to understand the processes that shape the evolutionary trajectory of clades. The repeated and similar large-scale morphological evolutionary trends of distinct lineages suggest that adaptation by means of natural selection (functional constraints) is the major cause of parallel evolution, a very common phenomenon in extinct and extant lineages. However, parallel evolution can result from other processes, which are usually ignored or difficult to identify, such as developmental constraints. Hence, understanding the underlying processes of parallel evolution still requires further research.

**Results:**

Herein, we present a possible case of parallel evolution between two ammonoid lineages (Auguritidae and Pinacitidae) of Early-Middle Devonian age (405-395 Ma), which are extinct cephalopods with an external, chambered shell. In time and through phylogenetic order of appearance, both lineages display a morphological shift toward more involute coiling (i.e. more tightly coiled whorls), larger adult body size, more complex suture line (the folded walls separating the gas-filled buoyancy-chambers), and the development of an umbilical lid (a very peculiar extension of the lateral shell wall covering the umbilicus) in the most derived taxa. Increased involution toward shells with closed umbilicus has been demonstrated to reflect improved hydrodynamic properties of the shell and thus likely results from similar natural selection pressures. The peculiar umbilical lid might have also added to the improvement of the hydrodynamic properties of the shell. Finally, increasing complexity of suture lines likely results from covariation induced by trends of increasing adult size and whorl overlap given the morphogenetic properties of the suture.

**Conclusions:**

The morphological evolution of these two Devonian ammonoid lineages follows a near parallel evolutionary path for some important shell characters during several million years and through their phylogeny. Evolution of some traits (involution, umbilical lid) appears to be mainly driven by adaptation to improve the hydrodynamic properties of the shell, whereas other characters (sutural complexity) evolved due to covariation with features that play a central role in the morphogenesis of mollusc shells. This example provides evidence that parallel evolution can be driven simultaneously by different factors such as covariation (constructional constraints) and adaptation (natural selection).

## Background

Independent evolution of similar biological traits in two different lineages branching off from the same ancestor defines parallel evolution [1-3]. It is a common phenomenon described for many animal clades (see e.g., [4-13]), including molluscs [1,14-19]. Repeated patterns of parallel evolutionary change of phenotypic traits are commonly regarded as evidence of adaptation under common selection pressures such as common environmental factors [20-22], therefore illustrating natural selection's major role in shaping morphological evolution and the repeatability of evolutionary processes. Several additional processes have been proposed that could contribute to the fabric of parallel evolution. However, the contribution and conditions in which these various processes trigger parallel morphological evolution are still insufficiently investigated. Furthermore, understanding the processes involved in parallel evolution is also important for solving systematic problems and thus to estimate evolutionary rates and diversity [14].

Evolutionary steps in two independent lineages will never be absolutely identical [1,15,16,23,24]. Nevertheless, morphological (phenotypic) evolutionary trends do occur in independent lineages and can display striking parallel changes (for a Recent mollusc example, see [17]). Here, the term parallel evolution is used in a broad sense, implying that not all parts of the organism undergo parallel evolutionary transformations and that the trends are nearly parallel, resulting in very similar organs/structures in a phylogenetic series of more than one species in at least two lineages.

Parallel evolution is often difficult to differentiate from convergence (evolution from two different stages of separate lineages toward an evolutionary stage that has evolved striking similarities among some phenetic, genetic or other traits; [25]) and some authors have even suggested a continuum between convergent and parallel evolution [2,26]. The distinction between convergent, parallel, and divergent evolution indeed requires the historical evolutionary aspect of studied lineages. Because it is the only direct evidence of evolution in the past over long time spans, palaeontological data can provide important insights into patterns and processes of parallel evolution.

In the fossil record, the Ammonoidea (Cephalopoda, Mollusca) are well-known to display large-scale morphological macroevolutionary trends [27-39]. These marine extinct cephalopods with an external, chambered shell have repeatedly been proven to be valuable study objects to develop or test evolutionary hypotheses [27,29,34-36,40-50]. Besides, ammonoids "are for palaeontologists what *Drosophila *is in genetics" [51]. Their usefulness in evolutionary biology originates in their high evolutionary rates, high taxonomic diversity and morphological disparity, and usually well-known stratigraphic (i.e. temporal) framework (see [52-54]). However, such morphological evolutionary trends among ammonoids have been rarely discussed and quantified in detail [38,39,47,55-58]. Knowledge of details in such lineages with seemingly "directed" morphological changes is of great interest, because not all evolutionary morphological changes in the ammonoid shell have the same causes. On the one hand, some evolutionary changes in ammonoid shell morphology may be constrained by covariation (e.g., Buckman's laws of covariation [59]; see discussion) and may thus be a result of constructional and/or developmental constraints. On the other hand, cases of parallel evolution of oxyconic shells (i.e. slender, compressed conchs with acute venter) in various lineages of ammonoids have been repeatedly documented and interpreted as adaptations to rapid and/or improved swimming (e.g., [58,60-63] and see discussion) and thus as a result of natural selection.

**Figure 1:**
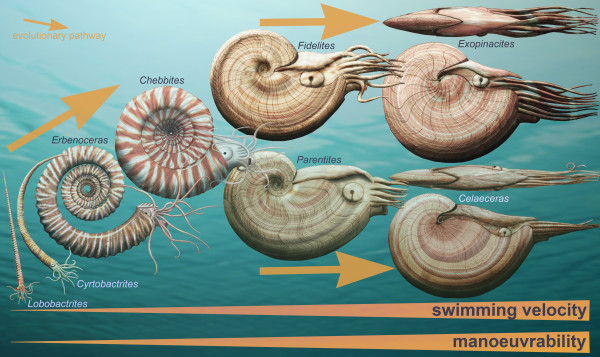
**Morphological evolution of externally-shelled cephalopods during the most intense phase of the "Devonian Nekton Revolution" in the Early and early Middle Devonian**. Reconstructions of the loosely coiled ancestors of the two lineages under consideration and two representatives of each of the lineages leading to and comprising the Auguritidae and Pinacitidae. The reconstruction of the soft-body are largely speculative: 10 arms are based on the knowledge that both plesiomorphic coleoids have ten arms and the sister group of the Ammonoidea + Bactritoidea + Coleoidea, the Nautiloidea, is known to begin with ten arms buds in early embryonic development [201]. The orientation can be reconstructed from the shell morphology [58]. Presence of a hood and of a camera lucida-style eye as in Recent Nautilida is speculative [158]. The position of the eye and the hyponome are deduced from the position of the ocular sinus and hyponomic sinus, respectively. The reconstructions are not shown at the same scale.

We here report on a probable, recently discovered case of parallel evolution among ammonoids. It happened very early in the history and rapid diversification of the Ammonoidea during the Early and Middle Devonian in the time interval between about 405 and 395 Ma [64]. The ammonoid shell, which grew by accretion, consists of a roughly conic, chambered, calcified, often ornamented and (more or less regularly) coiled conch. Yet, during this early diversification phase, the history of ammonoids is characterized by a morphological macroevolutionary trend from straight-shelled ancestors (bactritoids; for a review of cephalopod phylogeny, see [65]) via loosely coiled earliest ammonoids toward completely tightly coiled forms with closed umbilicus (Figure 1; [58,66-70]). This major evolutionary trend is thought to have occurred in a time interval of only about 2 My (see [58,69]). This rather fast evolution is evidenced by the co-occurrence of loosely coiled primitive forms, which are associated with contemporaneous, more derived, coiled forms in several localities that yielded the earliest ammonoids [71-78]. Furthermore, this morphological evolutionary trend occurred during the most intense phase of the "Devonian nekton revolution" [79]. This macroecological event corresponds to an explosive trend from planktonic and demersal marine animals toward true nekton as represented by the great diversification of jawed fish and ammonoids, reflecting a selection for swimming capabilities. It coincided with macroevolutionary transformations among various mollusc groups: an increasing proportion of gastropods formed tightly coiled protoconchs [80]; some dacryoconarids [81,82], ammonoids [58] and some nautiloids [83,84] more or less simultaneously evolved coiled shells, mainly during the Early Devonian. Among ammonoids, these post-embryonic morphological transformations enhanced buoyancy and swimming capabilities [58,85].

**Figure 2:**
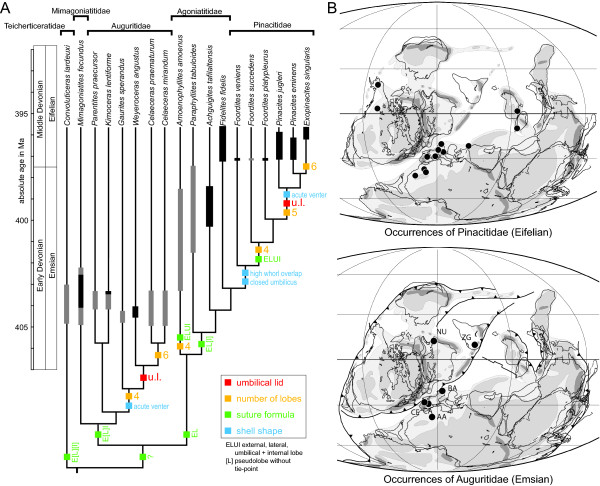
**Spatial and temporal distribution of Auguritidae and Pinacitidae in the Devonian**. A, Phylogeny and stratigraphic ranges of studied Devonian ammonoids. Stratigraphic ranges of taxa compiled from [74-77,88,91,93,202] and own unpublished data. The phylogenetic bifurcations are at an arbitrary stratigraphic position; thick vertical bars indicate temporal ranges based on fossil evidence (black = stratigraphic range after [77,88]; grey = stratigraphic range after literature, see text). B, Palaeogeographical maps (modified from [203]; see http://www.scotese.com) for the Early and Middle Devonian showing auguritid (bottom) and pinacitid (top) occurrences: AA: Anti-Atlas, Morocco; CA: Cantabrian Mountains, Spain; CE: Celtiberian Mountains, Spain; NU: Northern Urals; ZG: Zeravshan-Gissar Range, Uzbekistan.

**Figure 3:**
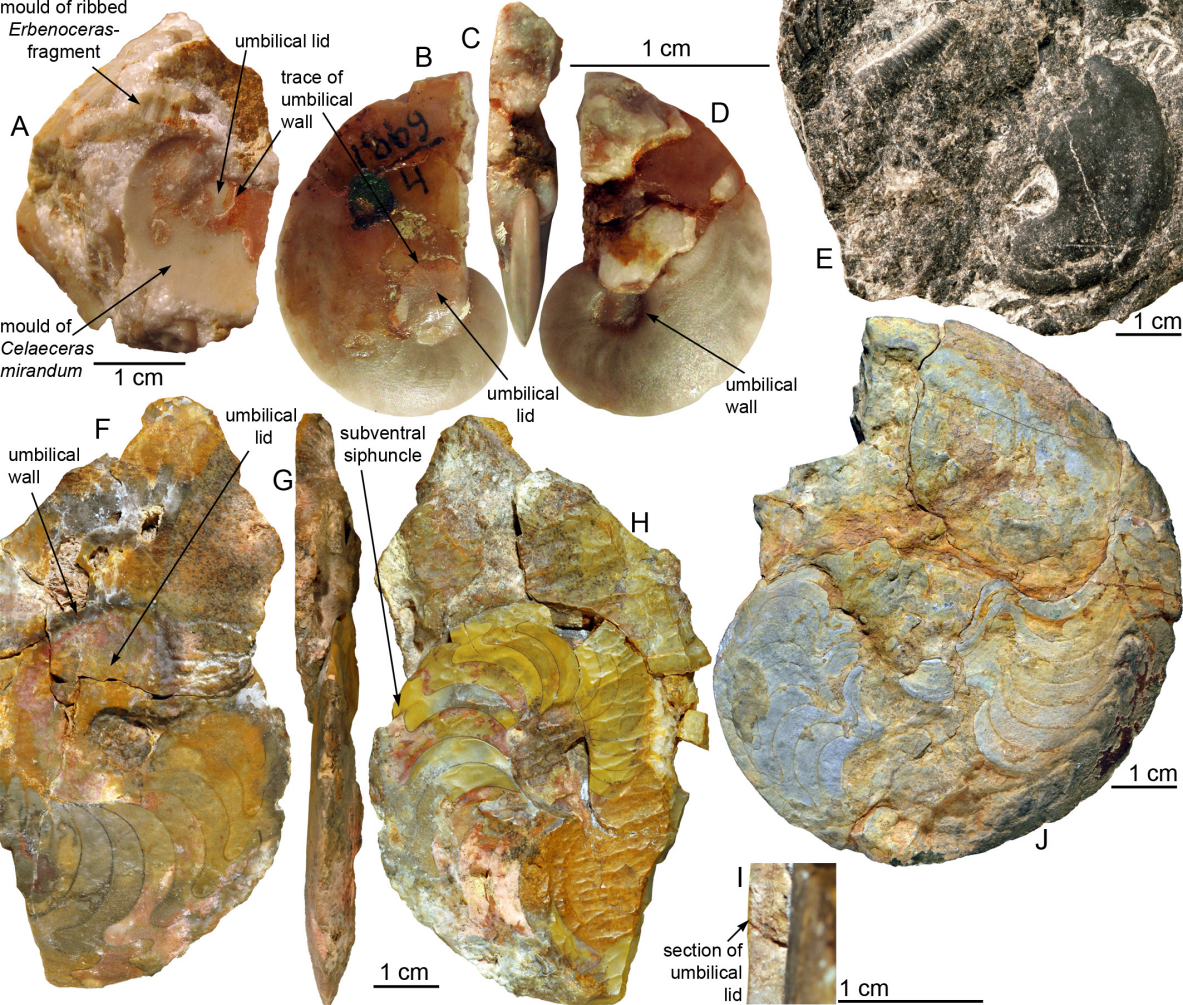
**Auguritidae and Pinacitidae showing the peculiar umbilical lid**. A-D, *Celaeceras mirandum*, PIN No. 1869/4, lower Emsian, North Urals, Russia. The specimen and its mould from both sides (B, D) and in apertural view (C). Note the mould of a whorl fragment of *Erbenoceras *or a closely related early ammonoid taxon on the top left in A, documenting the rapid evolution from loosely to tightly coiled ammonoids, which resulted in the co-occurrence of both morphological extremes. E, *Kimoceras lentiforme *associated with a fragment of a loosely coiled anetoceratid, PIMUZ 28869, lower Emsian of Shirdak Stow, Zeravshan, Uzbekistan. F-I, *Weyeroceras angustum*, PIMUZ 28449, lower Emsian, Bou Tchrafine, Tafilalt, Morocco. F, left side; note the broad yellowish umbilical lid. G, apertural view; note the slender oxyconic shell morphology and the relatively complicated suture line. H, right side; note the subventral position of the siphuncle. I, septal perforation (refigured from [73]). J, *Exopinacites singularis*, internal mould, PIMUZ 28866, middle Eifelian, El Kahla, Tafilalt, Morocco; note the relatively complicated ventral part of the suture line.

The case of parallel evolution studied here includes two families of ammonoids, namely the Auguritidae and Pinacitidae (Figure 2A). These two ammonoid lineages are of Emsian and Eifelian age (~ 405-395 Ma; [86]) and have a widespread palaeogeographical distribution (Figure 2B). Their stratigraphy and taxonomy have been revised recently [77,87-91]. Representatives of the older lineage are very rare [77,78]. The study of recently discovered material [78] and reinvestigation of the most complete material available in museum collections yielded comprehensive morphometric data and revealed the presence of an umbilical lid (see below) in the auguritid lineage, previously only known in the pinacitid lineage [92]. Both lineages under consideration share a common ancestor (which probably resembled *Convoluticeras lardeuxi*, Figure 2A) and their end-members have a very similar morphology in post-embryonic ontogeny. Although closely related and in spite of their great morphological resemblance, there is clear evidence for these two families being distinct clades. For instance, the embryonic shell was openly coiled in the auguritids [75,76,93] and became tightly coiled in later ammonoid evolution, including the pinacitids [88]; this is a character that was never reversed throughout ammonoid evolution, not even in Mesozoic ammonoids with uncoiled post-embryonic shells [45,94]. Additionally, the youngest representatives of the auguritids are at least 5 My older than the youngest representatives of the pinacitids. The evolutionary relationships of the auguritids and pinacitids were elucidated with a comprehensive cladistic analysis [89] that considered all currently known valid taxa (except *Achguigites tafilaltensis *[88] and *Weyeroceras angustus *[77], which were introduced later; for their phylogenetic position, see [95]). The resultant strict consensus is well resolved (Figure 2A). Both lineages are characterized at the end of their evolution by the development of a very peculiar morphological feature not known in this specific form in any other ammonoids (including forms with similar conch shapes). This peculiarity is the development of an umbilical lid (Figure 3), which is an extension of the lateral shell wall covering the umbilicus [92].

In the present ammonoid case study, we first describe and investigate the morphological evolutionary patterns of both families along their phylogenetic sequence (i.e. their phylogenetic order of appearance) with bivariate plots of quantitative characters of the ammonoid shell. Then, we identify and evaluate statistically the characters, which may have evolved in parallel and identically. Finally, we try to decipher the characters, which evolved identically because of covariation (constructional constraints) and/or because of adaptation (selective constraints).

## Methods

**Figure 4:**
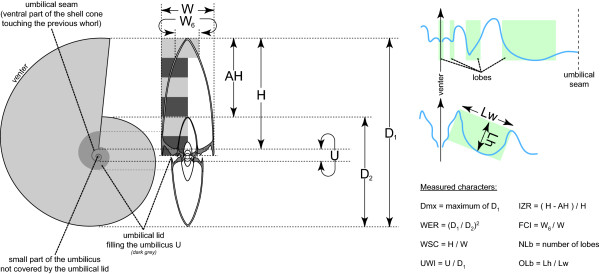
**Studied morphological parameters of the ammonoid shell**. Scheme showing the linear measurements used to characterize and quantify the morphological evolution of the ammonoid shell in this study (see text for further details).

To describe and analyze this case of parallel evolution of ammonoids, their shell geometry is here quantified by means of eight classical linear measurements, which characterize the major morphological features of the ammonoid shell (Figure 4; see also [47,96]).

•	The maximal diameter (Dmx) is the maximal shell diameter known for each species and is used to approximate the adult body size of the ammonoid species under consideration.

•	The whorl expansion rate (WER) is a measure of the proportional increase of shell diameter through growth (initially defined by [97,98], but we used the equation of [47], which is much easier to apply on actual specimens). It is considered one of the most important and biologically meaningful parameters because it roughly reflects the growth rates of the coiled shell tube and strongly correlates with body chamber length, soft part volume and the *syn vivo*-orientation of the shell [58,97-99].

•	The whorl shape compression (WSC) is a measure of the ellipsoid of the whorl section of the ammonoid shell aperture, which is a very important ammonoid taxonomic character due to the accretionary growth of the shell.

•	The umbilical width index (UWI) is the ratio between the umbilicus and the shell diameter and thus approximates the amount of shell coiling (degree of involution).

•	The imprint zone rate (IZR) describes the relative overlap of two succeeding whorls in terms of height.

•	The flank convergence index (FCI; modified after [100]) approximates the relative compression of the ventral part of the shell compared to its dorsal part (i.e. acute vs. low-arched rounded venter).

•	The number of lobes (NLb) approximates the indentation of the suture line, which is the junction line of the septa (chamber walls) with the internal side of the shell. Here, we counted only the lobes on one flank, including those in the plane of symmetry (i.e. internal and external lobes).

•	Last, the relative depth of the lateral lobe (= "O-lobe" of [101]; OLb), which is the ratio between width and height of the lateral lobe, measured from the apertural apices of the neighbouring saddles (see Figure 4).

All available specimens of the two families have been measured to quantify these characters. Most data are based on own measurements and some were taken from the literature [66,76,77,88,95]. The material referred to in this paper is housed in the following institutional collections: Palaeontological Institute, Moscow (PIN); Palaeontological Institute and Museum, University of Zürich (PIMUZ); National Museum, Prague (L 11705); Institute for Geosciences, University of Tübingen (GPIT).

The eight quantitative parameters are composed of one size measure (Dmx), six ratios (WSC, WER, UWI, IZR, FCI, OLb) and one ordinal count (NLb). For Dmx, FCI, NLb and OLb, only the adult value of each species is reported and not juvenile values, because these parameters always lie near structural boundaries at hatching (e.g., the suture is simple and will necessarily increase its complexity through growth; see discussion).

We here consider parallel evolution in a broad sense by assuming that not all characters are involved in the parallel evolution and by not assuming that evolutionary changes are accomplished by similar alterations in the developmental program (contra [2,102]). From these definitions, parallel evolution of some characters can be identified when the evolutionary trajectories of the studied lineages in the morphological space defined by this subset of characters (1) start with the same morphotypes, (2) evolve in parallel and are overlapping, and (3) end with the same forms. In other words, the evolutionary trajectories are identical in origin, magnitude and direction. This pattern of parallel evolution must be distinguished from parallelism in phenotypic space. This different phenomenon concerns lineages having parallel trajectories (same direction), but not necessarily the same origin and/or magnitude (for an example of parallelism but not parallel evolution, see [103], p. 828, figure 3C). It has to be taken into account that the likelihood of (1) finding statistical support of parallel evolution as well as of (2) parallel evolution itself to occur is dramatically reduced when the evolutionary transformations change more often in several aspects (direction, quality, quantity, proportion) in both lineages. Simple cases of parallel evolution are thus easier to test but less meaningful with respect to selective forces and vice versa.

**Figure 5:**
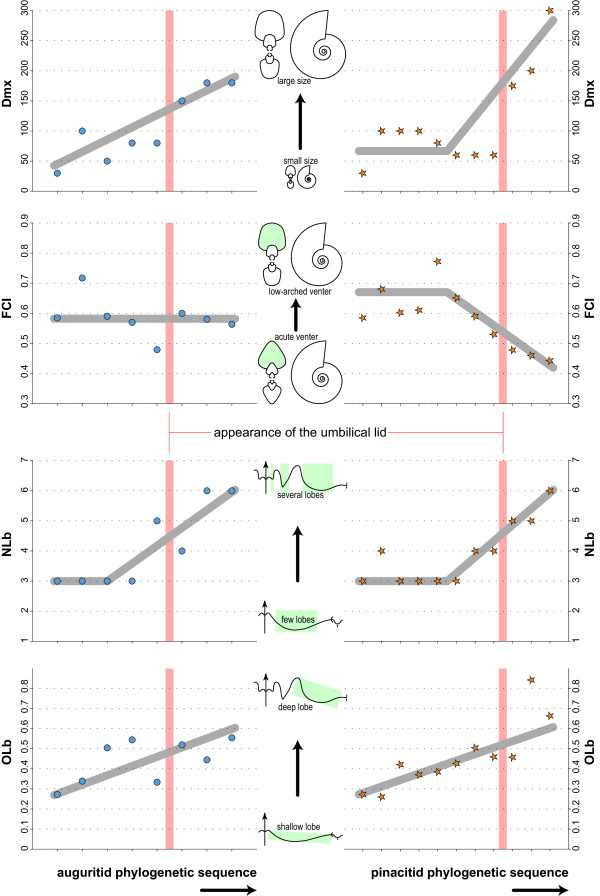
**Evolution of Dmx, FCI, NLb and OLb in auguritids and pinacitids**. Bivariate plots of the evolution of adult size, flank convergence index, number of lobes and relative depth of the lateral lobe through the phylogenetic sequence of the two studied ammonoid lineages, separately. Grey line indicates empirical evolutionary trend.

**Figure 6:**
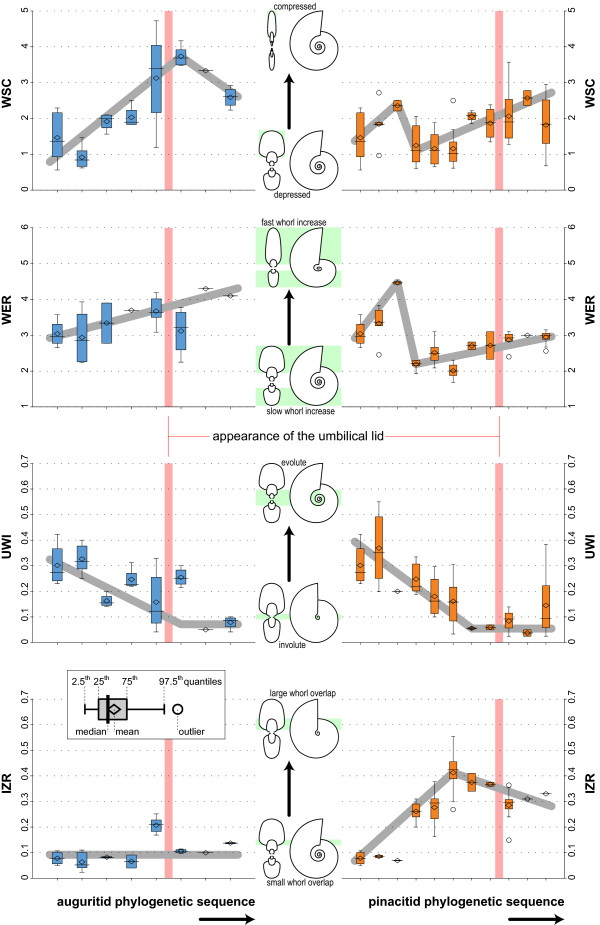
**Evolution of WSC, WER, UWI and IZR in auguritids and pinacitids**. Box plots of the evolution of whorl shape compression, whorl expansion rate, umbilical index and imprint zone rate through the phylogenetic sequence of the two studied ammonoid lineages, separately. Grey line indicates empirical evolutionary trend.

**Figure 7:**
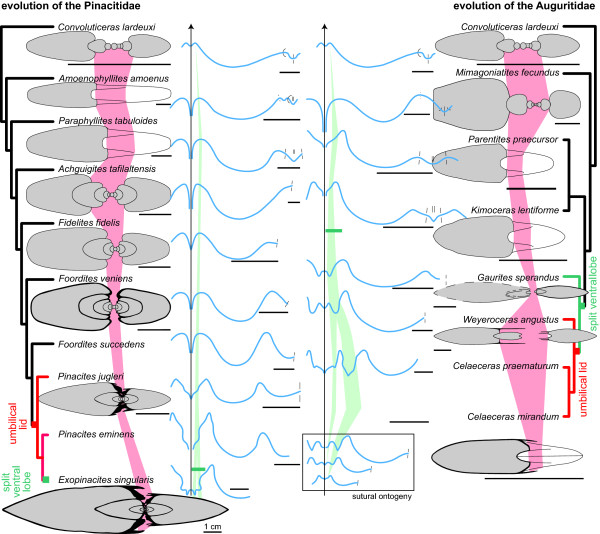
**Outlines of shell whorl section and suture line for Auguritidae and Pinacitidae**. Cladograms of the lineages leading to, and including, the Eifelian Pinacitidae and the Emsian Auguritidae. Where available, a sketch of the cross section and a suture line drawing is given. Note the parallel trend toward oxyconic shells with acute venter, with umbilical lids, and with increasing numbers of sutural elements at venter and near the umbilicus divided by a broad lobe. Sections and sutures redrawn or taken from [66,73,76,77,88,92,93]. Sections of *Foordites veniens, Weyeroceras angustum *and *Gaurites sperandus *newly drawn after PIMUZ 28867, PIMUZ 28449 and PIN No. 3981/22 from the lowermost Eifelian of Hamar Laghdad, Morocco and the lower Emsian of Bou Tchrafine, Morocco and Yusupkul Stow, Uzbekistan, and that of *Celaeceras mirandum *from the lower Emsian of the North Urals, Russia, after PIN No. 1869/4. Scale bars all at 1 cm length.

**Figure 8:**
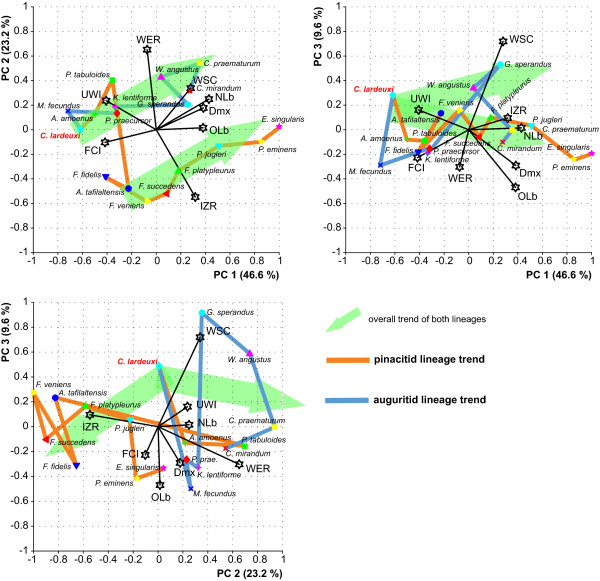
**Multivariate analysis of the eight measured shell parameters**. Results of the principal component analysis (PCA) based on the eight measured shell parameters. The PCA was done on a correlation matrix. The first three principal components axes account for about 80% of the total variation in shell morphology. Vectors of shell characters (length and direction compared to the PC axes) revealed that variation on PC1 is mainly associated with Dmx, WSC, NLb/OLb, FCI and UWI, PC2 with WER and IZR, and PC3 with WSC.

Before evaluating the parallel evolution of auguritids and pinacitids, we describe the patterns of morphological variation and evolution of these two lineages (Figures 5, 6, 7, 8). Patterns of morphological evolution are examined globally by means of a multivariate analysis based on the eight studied quantitative characters of the ammonoid shell (Figure 8). We perform a principal component analysis (PCA; [104]) to examine the variation of the variables within the sample and identify the characters that contribute to observed evolutionary changes by creating high variation. Since the studied characters are of different types (size, ratio, ordinal), the PCA has been performed on the correlation matrix (data standardized to mean zero and unit standard deviation) for all characters. Then, we examine the evolution of each quantitative shell character separately by means of bivariate plots depicting their distribution through the phylogenetic sequence for the two ammonoid lineages separately (Figures 5 and 6). These plots enable an empirical evaluation of the presence or absence of directed evolutionary changes (trends) for each character. Bivariate and multivariate exploratory analyses are performed by means of the versatile palaeontological data analysis freeware PAST ([105,106]; http://folk.uio.no/ohammer/past), as well as by scripts programmed by C.M. in MATLAB^® ^(http://www.mathworks.com/).

**Figure 9:**
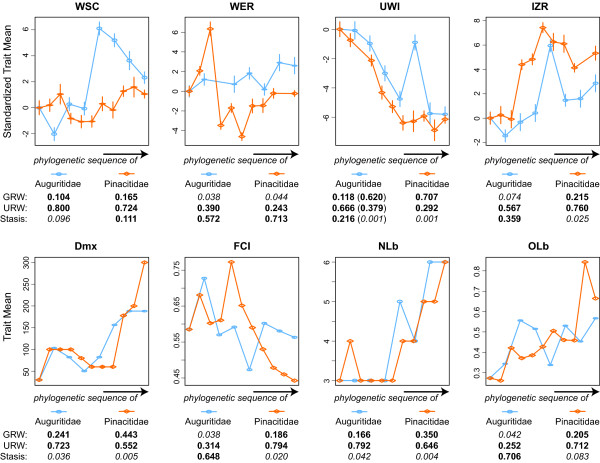
**Evolutionary trajectories for Auguritidae and Pinacitidae and their Akaike weights for three evolutionary models**. Standardized evolutionary trajectories of WSC, WER, UWI, and IZR and raw evolutionary trajectories of Dmx, FCI, NLb, and OLb for auguritids and pinacitids. The three tested evolutionary models are directed trends (GRW, general random walk), random trends (URW, unbiased random walk), and stasis (for details of the method, see text and [116]. Relative Akaike weights for models with more than minimal support are in bold, otherwise in italic. The most supported trends are those shared by both families (UWI, Dmx, and NLb) and characterize the parallel evolution of the two studied Devonian ammonoid lineages. For auguritids, the evolutionary changes for UWI initially weakly support the hypothesis of a directed trend, but if the outlier taxon (*Weyeroceras angustus*) is removed, this hypothesis is significantly supported (values in brackets).

Since apparent trends in evolutionary series can be produced randomly [107-113], the previously and empirically identified evolutionary trends are tested statistically. Several methods exist, which are based on random walk models, to test and characterize observed trends and to distinguish the three modes of evolutionary change commonly considered in palaeontological studies: directional change (GRW, general random walk), random walk (URW, unbiased random walk), and stasis [113-116]. The evolutionary changes of each character are here evaluated by means of the maximum likelihood method of [116-119]. The method is recognized to perform well even when evolutionary sequences are incompletely sampled, which is likely for empirical palaeontological sequences as documented here [116]. It has been implemented as a package (paleoTS, [116]) in the freely available statistical environment R (http://www.r-project.org/). The method evaluates the maximum likelihood of producing the observed trends for three evolutionary modes (GRW, URW, and stasis). The relative support of each of these three models is assessed using well-established statistical means such as Akaike weights ([120]; for details, see [116-118]), which indicate the relative likelihood for each of the three evolutionary models (Figure 9). Since auguritids and pinacitids branched off from the same origin, the characters displaying directed trends shared by both lineages and supported by the statistical analysis can *potentially *participate to a case of parallel evolution (Figure 9).

In order to assess the parallel evolution of the two studied lineages, we use two different approaches, both based on a multivariate analysis using the subset of characters previously identified to be potentially involved in this case of parallel evolution. Note that univariate approaches can suffer from a "dimensionality bias" and similarities of trajectories in a morphospace should preferably be tested multivariately [121,122]. First, the parallel evolution of this subset of characters is evaluated by means of a method developed for comparing evolutionary trajectories of phenotypic change [123]. According to this method, the phenotypic evolution of a lineage is defined as a trajectory across a set of evolutionary levels in a multivariate morphological space. Attributes of these trajectories (magnitude, direction and shape) are quantified and statistically compared across pairs of taxa by means of a residual randomization permutation method [123,124], and a summary statistic is used to determine the extent to which patterns of phenotypic evolution are concordant. Note that the method currently requires that the compared trajectories have the same number of evolutionary levels (i.e. in our case the same number of species). Since more species of pinacitids have been described, the analysis is performed by first merging the data of the phylogenetically closest species of the pinacitid lineage in order to obtain the same number of studied evolutionary levels or steps for both families. The two species of *Pinacites *have thus been merged, as well as *Foordites succedens *with *F. platypleurus *and *Achguigites *with *Fidelites*. This constraint of the method reduces the power of the test since the species of different lineages cannot be considered as equivalent.

The second method to test the parallel evolution of these Devonian ammonoids follows the approach proposed by [121] for comparing ontogenetic trajectories. This method is a permutation test based on within-lineage multivariate regression of the characters hypothesized to be involved in the parallel evolution. If the two lineages evolved in parallel, then their phylogenetic trajectories are identical in the morphological space defined by the subset of characters involved. To test this hypothesis, we first compute for each lineage separately a linear total least square regression, then we sum the squared orthogonal distance for each specimen from its nearest point on the regression curve. This sum provides the original test statistic for subsequent comparison. Then, we randomly resample without replacement a large number of times the taxonomic assignment of studied specimens to the two lineages and recompute the summed squared distances of these permuted families (this provides the permutation distribution). If the two studied lineages evolved in parallel, the original test statistic should not be an outlier in the permutation distribution of summed squared distances (see [121]). In other words, permuting specimens' affiliation does not increase the residuals of the multivariate regressions and this is possible only if specimens of both families are close together in the studied morphological space.

## Results

### Evolution of Auguritidae (Early Devonian)

The evolution of shell characters through the phylogenetic sequence of the lineage that includes the Auguritidae is described and reported in Figures 5, 6, 7 and 8. Within this lineage, several evolutionary trajectories can be empirically suggested: the adult shell size (Dmx), whorl shape compression (WSC), number of lobes of the suture (NLb) and relative depth of the lateral lobe (OLb) increase simultaneously with decreasing umbilical diameter (more tightly coiled shells; UWI). The whorl expansion rate (WER) also increases slightly. The flank convergence index (FCI) and imprint zone rate (IZR) fluctuates without emerging trends.

Of great interest is the general trend of increasing involution (more tightly coiled shells as shown by the decreasing UWI, Figure 6). Indeed, most ammonoid specimens of lower Emsian age are very openly coiled and share a wide umbilical perforation [45]. It is striking that all forms of this lineage are still associated with loosely coiled Anetoceratinae (Figure 3E; see references in the background section), which indicate that their evolution must have been comparably fast. In addition to this trend toward a closed umbilicus, the lineage is characterized by the appearance of an umbilical lid. Although the most derived forms of this lineage are rather rare, re-examination of the material from the Urals (Russian) and the Zeravshan Range (Uzbekistan) in the Palaeontological Museum of Moscow [75,93] and new specimens from Morocco [78] reveals that the two most derived genera *Weyeroceras *and *Celaeceras *both possess extensions of the lateral shell wall covering the umbilicus (i.e. umbilical lid; Figures 3, 7). This structure was previously known in this form only from the pinacitids [92]. Noteworthy, the trends toward more compressed (WSC) and more involute (UWI) shells levelled off with the appearance of the umbilical lid. The appearance of the umbilical lid is also associated with the smallest UWI (almost closed umbilicus).

### Evolution of Pinacitidae (Early and Middle Devonian)

The evolution of shell characters for the lineage that includes the Pinacitidae and which evolved from the same remote ancestor of auguritids is reported in Figures 5, 6, 7 and 8. Within this lineage, the adult shell size (Dmx), the number of lobes of the suture (NLb) and their relative depth (OLb), as well as the acuteness of the venter (FCI) increased simultaneously, especially among the more derived species. The umbilical width index (UWI) and the imprint zone rate (IZR) also display trends but these occur only among the more primitive species. Evolutionary changes of whorl shape compression (WSC) and whorl expansion rate (WER) display different, slightly more complex evolutionary patterns: a quick increase in the most primitive species, an abrupt reset and then a slight increase in the most derived species, giving the trend a sigmoid course.

This lineage is better known, much more abundant and more diverse than auguritids. The pinacitids had a nearly cosmopolitan distribution during the Middle Devonian (see Figure 2B; [87,88]) and even the most derived representatives were locally quite common. Like in the auguritids, the derived species of pinacitids acquire more oxyconic shells and more complex sutures, as well as an umbilical lid. With the appearance of the umbilical lid, the trends toward greater involution (decreasing UWI) and whorl overlap (IZR) levelled off (Figure 6). This levelling off may correspond to a "left-wall" effect, i.e. the trend cannot go further once the umbilicus is closed, because the closure of the umbilicus marks a constructional boundary (whorls completely overlap).

### Parallel evolution of the two lineages

The results of the morphological principal components analysis are plotted in Figure 8. The first three components extracted from the dataset accounted for 79.4% of the morphological variation. The PCA plots indicate which shell characters contribute to the morphological evolution of Devonian ammonoids by creating high variation. Vectors of shell characters (length and direction compared to the PC axes) revealed that variation on PC1 is mainly associated with Dmx, WSC, NLb/OLb, FCI and UWI, PC2 with WER and IZR, and PC3 with WSC. When viewed in the PC1/PC2 plot (Figuer 8), the morphological evolutionary trajectories of Auguritidae and Pinacitidae display a seemingly pattern of parallelism. However, this pattern is absent on other PC axes and is thus an artefact of projection onto a reduced number of axes (compare figure 5 in [121]). Noteworthy, it does not reflect the case of parallel evolution discussed here, since this PCA is based on the eight quantitative characters and not only those really evolving in parallel (see below). Finally, this principal component analysis shows that the two studied clades are clearly distinct morphological clusters even if related to a common ancestor, because they occupy distinct areas of morphospace. Therefore, even if these lineages experience a parallel evolution of some characters (see below), each lineage is clearly distinct and has its own evolutionary history. The auguritid lineage has also a more irregular evolutionary trend than the pinacitid clade. This higher inter-specific variation probably originated from the poorer database and the fewer evolutionary steps for this rare family.

Both ammonoid lineages display empirical morphological evolutionary trends of some shell characters. Their statistical evaluation by means of the method of [116] is illustrated and reported in Figure 9. Among the three tested evolutionary patterns (directed trend, GRW; random trend, URW; stasis), the studied quantitative characters are mainly characterized by random trends and/or stasis (Akaike weights of URW or Stasis > 0.5). The only well-supported directional trend (GRW > 0.5) is for UWI (increasing involution) in the pinacitids, as well as in the auguritids if we remove one "outlier species" (Figure 9). Hence, this suggests that, except for UWI, the two ammonoid lineages have no directed evolutionary changes of their morphology. However, we must acknowledge that the power of this statistical test is reduced by the current state of our data. First, although we managed to acquire a comprehensive dataset from a palaeontological point of view, the number of species and specimens in the studied dataset remains low. Second, the studied ammonoids display important ontogenetic changes [88,125], which largely increase the variance and range of studied characters. However, the scarcity of well-preserved, adult specimens of these earliest ammonoids prevents using only adult values. If we relax the necessary statistical support, two studied shell characters may display possible directed trends for both lineages: Dmx and NLb, which have negligible values for stasis and low but not negligible support for GRW. By comparison with other ammonoid groups [28,29,31-33,35,36,39], trends in these two characters are expectable, but it remains to be tested by additional material in the future. Nevertheless, all other characters remain devoid of directional trends. Thus, the two studied lineages share the same directed trends for UWI with certainty, and more hypothetically for Dmx and NLb. Finally, it also appears plausible that the evolutionary trajectories for these three characters end with forms having equal means: for UWI, the last auguritid and the last pinacitid have no significant difference in their mean (Welch *t *= -1.588, *p *= 0.139); for Dmx, the last auguritid has the same value has the penultimate pinacitid but the last pinacitid is larger than the last auguritid; and for NLb, both lineages have at the end the same number of suture elements. This is also suggested graphically in Figure 9.

**Figure 10:**
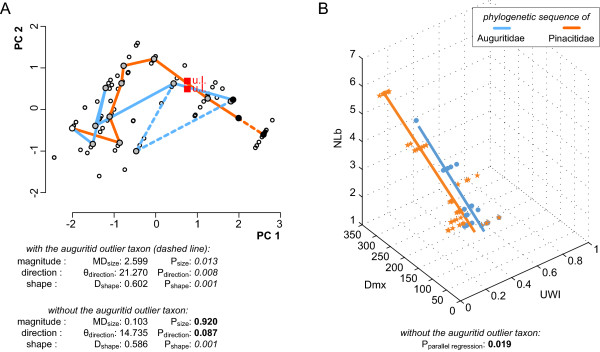
**Evolutionary trajectories of Auguritidae and Pinacitidae in the morphological space defined by the three characters involved in the case of parallel evolution**. A, Statistical evaluation of the parallel evolution by means of the trajectory approach of [123]. Plot of the first and second principal components estimated from the correlation matrix for auguritids and pinacitids based on the three standardized characters UWI, Dmx, and NLb. Statistical evaluation of the parallel evolution is indicated with and without the auguritid outlier taxon *Weyeroceras angustus *(dashed line). Parameters with more than minimal support are in bold. There are no significant differences in magnitude and direction of the trajectories, but they are different in shape. B, Statistical evaluation of the parallel evolution by means of the regression approach of [121]. The phylogenetic trajectory of each lineage is fitted by a linear total least square regression. The statistical evaluation is indicated without the auguritid outlier taxon. The *p*-value of the test (*p *= 0.0197) is low, but the hypothesis of parallel trajectories could not be rejected by the permutation test.

Since auguritids and pinacitids originated from the same ancestor, had possible directional trends in UWI, Dmx and NLb, and finally ended with similar values for these three characters, auguritids and pinacitids may have a parallel evolution for three quantifiable traits (coiling, adult size, and suture complexity). There is, however, also the evolution of the umbilical lid, which is a presence/absence character and does not influence the quantitative results. The hypothesis of parallel evolution of the measurable characters is tested by two permutation methods based on the character subset made by UWI, Dmx and NLb (Figure 10). Using the trajectory approach of [123], it appears that there are no significant differences in the magnitude (MD_size _= 0.103, *P*_size _= 0.920) and in the direction (θ_dir _= 14.735, *P*_dir _= 0.087) of phenotypic evolution between the two lineages (Figure 10A). However, there are significant differences in the shape of the two evolutionary trajectories (D_shape _= 0.586, *P*_shape _= 0.001). This difference in the shape of the two trajectories is, however, expected because the taxa of each lineage are not truly equivalent and do not necessarily represent the same evolutionary steps.

Using the regression approach of [121], it appears that the hypothesis of parallel trajectories of the two studied lineages cannot be rejected (*p *= 0.019), but the value is low (Figure 10B). The statistical evidence for the parallel evolution in UWI, Dmx and NLb of auguritids and pinacitids is thus controversial between the two approaches we used. As discussed previously, our sparse and unbalanced dataset is probably responsible for the low power of these statistical tests. Additional data are thus required to better test this hypothesis of parallel evolution. Noteworthy, both lineages developed the peculiar umbilical lid, not known in any other ammonoid group. It still appears likely that auguritids and pinacitids evolved in parallel with respect to the increasing involution, adult size, suture complexity and evolution of an umbilical lid.

In summary, this probable but not fully proven case of parallel evolution included the following quantitative and qualitative traits and their corresponding evolutionary trends (Figuers 5, 6, 7, 8 and 9): (1) adult body size (Dmx: from less than 50 mm to more than 150 mm for auguritids and 300 mm for pinacitids); (2) umbilical width (UWI: from moderately wide to closed; and with formation of an umbilical lid by extending the lateral shell wall across the umbilicus); (3) sutural complexity (NLb: from simple to more complex by ventral and umbilical insertion of accessory elements); (4) siphuncle position (not quantified: from ventral to subventral, see e.g. Figuer 3H); (5) aperture shape/whorl cross section (from platycone to oxycone, i.e. more compressed shell and/or more acute venter); and (6) shape of venter (not quantified: from rounded to acute; Figuer 7). These trends occurred in a phase that lasted ca. 10 My from the last common ancestor of both lineages to the extinction of the last representative of the pinacitids. As shortly discussed in the introduction, parallel evolution is never exact and never includes all traits of the members of the lineages under consideration. Differences between the two ammonoid lineages can be seen in the following traits for example (Figuers 5, 6, 7, 8 and 9): (1) adult body size (Dmx: both increasing, but pinacitids reach over twice the size of the auguritids); (2) whorl overlap (IZR: trend and higher values for pinacitids); (3) suture line (higher values of OLb for pinacitids); and (4) aperture shape/whorl cross section (both from platycone to oxycone, but by increasing WSC for auguritids and increasing FCI for pinacitids).

## Discussion

Parallel evolution could theoretically have various causes such as chance, genetic heritage, covariation/developmental constraints, and/or adaptation/selection. In the context of cases of protracted parallel evolution of more or less complex structures or organs, which comprise many taxa in the lineages under consideration, the likelihood is very low that this parallel evolution is a result of sheer chance. Therefore, it is necessary to determine which causes contributed how, and to what degree to the documented evolutionary parallel and identical trends. In the case of auguritids and pinacitids, covariation of some traits is evident while we can suggest adaptation for other morphological aspects of the shell.

### Functional traits (adaptation)

Among the documented parallel morphological evolutionary trends, the most important are: increasing involution (more tightly coiled whorls toward a closed umbilicus); development of an oxyconic shell (more compressed shell and/or more acute venter); larger shell diameters (body size); and the terminal acquisition of an umbilical lid.

The increasing body size of both studied lineages constitutes an example of Cope's rule, known as the widespread tendency of animal groups to evolve toward larger body size [126-129]. This type of trend has been attributed to certain advantages of size increase such as increased defence against predation, increased food competition, increased success in mating, increased individual longevity, and better energy use [20,126-128,130-132]. Since the lineage starts with small shells, the observed trend toward a larger shell size conforms to the traditional gradualist and adaptive interpretation that large-scale evolutionary trends result from persistent selection within long-ranging lineages. Several authors have illustrated examples of increasing ammonoid shell size during initial radiation of a group [46,129,133,134]. However, [135] found no evidence for Cope's rule among Early Jurassic ammonoids, after the initial recovery radiation following the Triassic/Jurassic boundary extinction.

The persistent increasing involution (i.e. more tightly coiled whorls toward a closed umbilicus) coupled with the oxyconic shell as displayed by the auguritids and pinacitids is surely one of the most frequently observed large-scale morphological evolutionary trends among ammonoids [27,28,31-33,38,39,66,136-139]. This highly recurrent trend among numerous and distantly related ammonoid clades (thus independent of phylogeny) suggests that it may have a strong adaptive significance due to functional constraints [140,141]. Since ammonoids are an extinct group, we have no direct evidence of the cause and advantage of this possible adaptation. However, thanks to mechanical experiments on shell models and analytical calculations of shell hydrodynamics [61-63,99,142-144], as well as by analogy with Recent nautiloids (the only extant cephalopod with a chambered external shell; see [145,146]), numerous studies evaluated the hydrodynamic performances (locomotion) of the ammonoid shell shape. It has been widely demonstrated that, for shells with oxyconic shell shapes, the energy consumption for swimming is the lowest and potential maximal swimming speed is the highest. Manoeuvrability is best with roughly horizontal apertures at relatively short body chambers and more or less high whorl expansion rates [58,60,62,63,147]. Indications for a link between water energy, facies and conch form are not rare but only few publications on that matter are available [55,57,88,139,148-155]. Increased involution of the shell (decreased UWI) therefore appears to represent a consistent adaptation toward improved hydrodynamic properties of the shell (decreased drag, increased streamlining) and consequently probably improved predation efficiency, increased food competition, increased predator escape, and/or improved search for mating partners and suitable spawning regions. Hence, the parallel and identical trend of ammonoid shell involution in the Auguritidae and Pinacitidae is here suggested to be best and most plausibly explained by functional adaptation.

Finally, both studied lineages evolved a peculiar morphological trait, the umbilical lid. This shell modification occurred only among the most derived species of both studied evolutionary lineages. This umbilical lid represents an extension of the lateral shell wall, passing across the umbilical shoulder toward the coiling axis and consequently more or less completely occludes the umbilicus of the ammonoid shell. This construction of the umbilical lid is a unique feature of the two studied lineages and is not known in this form from any younger ammonoid group, not even in groups having similar shell shapes. Other ammonoid groups do occasionally possess morphological features occluding the umbilicus [92,95,156], but in these cases, the structure is only superficially similar and results from a different origin (e.g., thickened umbilical walls and/or reduced umbilical width [92]). Since there are countless taxa with an overall shell shape (oxyconic shell with a very narrow umbilicus) roughly resembling that of auguritids and pinacitids, the appearance of this morphological structure (the umbilical lid) cannot be simply explained by covariation, thus making an adaptive explanation most likely. The parallel evolution of umbilical lids could be functionally explained by different, not mutually exclusive hypotheses.

(1) The umbilical lid could represent an adaptation for the improvement of the hydrodynamic properties of the shell. Indeed, it has been shown repeatedly that the size and shape of the umbilicus in combination with the overall shell geometry has a profound influence on the hydrodynamic properties of the shell [57,61-63,142]. For instance, forms with a closed umbilicus reduce added mass (such as the water trapped by the umbilicus) and confer better acceleration and deceleration during swimming [63]. In this context, this hypothesis supports the adaptation to improve swimming, but it does not explain completely why the umbilical lid is constructed in such a peculiar way in only these two groups.

(2) In addition to the previous hypothesis, the umbilical lid could act as a device directing water into the mantle cavity from behind when swimming backwards by means of jet propulsion. Indeed, most cephalopods swim "backwards" by taking in water into the mantle cavity and by expelling this water by mantle cavity compression through the hyponome (e.g., [157]; for a discussion on the functional analogy between ammonoids and *Nautilus*, the only extant cephalopod with an external shell, see [63,146]). Note that all recent and all fossil cephalopods still have or had a hyponome [158]. This is corroborated by the presence of a hyponomic sinus in our forms just like in *Nautilus *[146]. In this case, the umbilical lid has hydrodynamic advantages because the water enters from the swimming direction and leaves in the opposite direction (i.e. in the direction of the aperture, where the animal came from) with a deviation through the mantle cavity where it is accelerated by the animal's musculature (Figuer 11). Furthermore, the umbilical lid is not formed at the beginning of ontogeny but rather at a diameter of about 5 mm. This roughly coincides with the size when swimming movements become more effective in the course of ammonoid development [62,63], thus supporting a link between shell hydrodynamics and the evolution of the lid. True evidence for this function is missing, but both lineages lived in a time when a general increase in mobility among swimming animals occurred [79].

**Figure 11:**
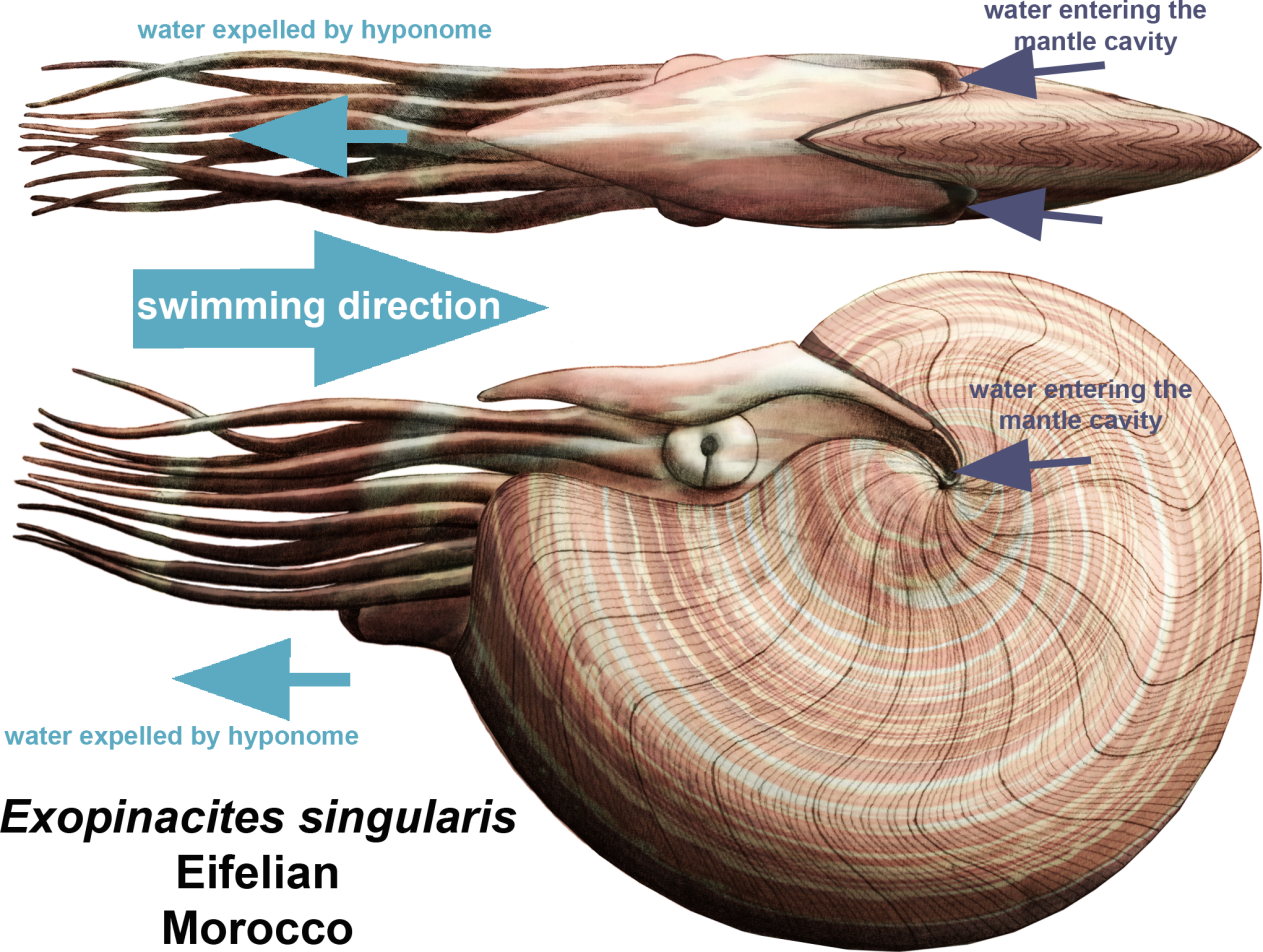
**Reconstruction of *Exopinacites singularis *and of its possible swimming advantage involved by the presence of an umbilical lid**. Speculative functional advantage of the umbilical lid, which was combined with a deep umbilical sinus in the aperture, allowing water to enter directly from the swimming direction into the mantle cavity. The position of the hyponome can be deduced from the hyponomic sinus.

(3) Finally, the umbilical lid might have been caused by, or linked to, features unrelated to the hard parts (e.g., soft-tissues, genes). For instance, the parallel evolution of an umbilical lid may speculatively result from the repetitive loss of expression of regulatory Hox genes (e.g., [159]; for the description of such a case in the evolution of crustacean maxillipeds, see [7]) or the repeated selection of developmental genes (see e.g., [10,160]). Logically, this is impossible to test on Devonian fossils. Furthermore, the genetic underpinnings of parallel and convergent evolution are generally unknown in Recent organisms [161]. Although it is not yet possible to find evidence for another function of this lid, it appears likely that it served indeed as a structure to improve swimming abilities.

### Covarying traits (constructional constraints)

Among the documented parallel morphological evolutionary trends, some can be explained as covarying traits, meaning that some morphological trends can result from constructional constraints [39,162-165]. The characters involved in such indirectly triggered trends are important to identify because, in this case, there is no need to search for an adaptive or genetic explanation.

Covariation of shell characters is well-known for ammonoids [166]. For instance, the intraspecific variation of an ammonoid species is usually expressed by the following gradient: the more evolute the shell, the thicker the whorl shape, and the more robust the ornamentation. It is referred to as Buckman's first law of covariation [59,163,167,168] and has been abundantly documented and discussed (e.g., [38,169-174]). Shape and differentiation of suture lines (i.e. the kind and degree of folding of the phragmocone chamber walls) also covary with shell shape and shell size (Buckman's second law of covariation). More precisely, the number of suture elements (frilling of the suture line) increases with the size of the shell (whorl height) and/or with the compression of the shell. This is evidenced by the widely documented increase in the suture complexity through ontogeny of the ammonoid shell (for Devonian taxa see, e.g., [47,66,93]; see also inside frame of Figure 7). Furthermore, both patterns are usually linked because in most ammonoid ontogenies, a more or less rapid change from more circular to either compressed or depressed apertures happened (see Figure 7). During this change in whorl section, the relative number of suture elements changes in such way that less suture elements occur when the whorl cross section is closer to a circle, and vice versa [51,175]. Both patterns are abundantly documented for all ammonoid groups (e.g., [44,47,55,60,66,93,95,176-184]).

The function of the septal folding is the subject of much debate (e.g., [150,185,186]) and many hypotheses have been proposed, such as buttressing [144,187,188], muscle attachment [189], cameral liquid transfer [190], metabolic effect [191], developmental epiphenomena [175,192-194], and/or locomotion [195]. Nevertheless, the increase with size and through ontogeny is expected because septal formation behaves like a "viscous fingering" phenomenon (see review of [175]); in this morphogenetic model, the details (not the general outline) of the suture pattern depend on the space and shape available for the suture during its formation ("domain effect").

**Figure 12:**
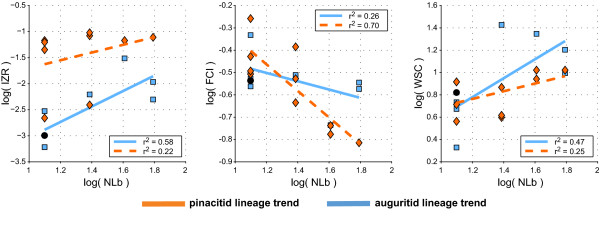
**Covariation of suture line geometry and shell geometry**. Bivariate plots of IZR, FCI and WSC versus number of lobes (NLb); the number of lobes increased throughout the phylogeny of both lineages.

Both studied lineages are no exception to this rule of covariation. The two lineages are characterized by the parallel and identical evolution toward an increasing number of lobes (Figure 5). In the case of pinacitids and auguritids, new lobes and saddles were especially inserted dorsally and ventrally, where the shell void was the narrowest (Figure 7). Note that in the two derived species *Weyeroceras angustum *(Auguritidae) and *Exopinacites singularis *(Pinacitidae) the siphuncle shifted to a subventral position causing the formation of an additional lobe [78,92]. Both lineages are also characterized by an increasing adult size and an increasing oxyconic character of the shell (more compressed shell and/or more acute venter; Figures 5 and 6). Hence, in the case of these Devonian ammonoids, the trends toward increasing number of suture line elements are likely to be induced by covariation as corroborated by the strong correlation of NLb with IZR, FCI and WSC (Figure 12). This covariation of NLb with WSC and FCI is not the indirect result of parallel evolution, because the two studied lineages display different trends for WSC and FCI (neither parallel, nor identical; Figures 5, 6, 9). Hence, both studied lineages experienced a parallel and identical trend toward more complex suture patterns, both by covariation with other evolutionary trends of the shell shape, but this is achieved differently in both lineages (mainly WSC for auguritids and FCI for pinacitids).

## Conclusions

Very early in the course of the evolution of the Ammonoidea, the two families Auguritidae and Pinacitidae evolved independently but display a striking pattern of probably parallel morphological evolution. These two families share several essential morphological traits such as shell shape, suture line course, and the presence of an umbilical lid, which is an extension of the lateral shell wall and is unknown in this form from any other ammonoid lineage. The similar and parallel evolution of both lineages toward large, involute shells with more complex suture lines and with closed umbilicus including the formation of an umbilical lid can be explained best by selection for enhanced hydrodynamic properties of the shell (selective/adaptive constraints). Speculatively, the umbilical lid might have facilitated the intake of water from the swimming direction during repulsion swimming. Some other shell parameters also show a parallel evolution, but most likely due to covariation (constructional constraints). The increase in sutural complexity represents a typical case of covariation induced by the evolution toward involute and larger shells with acute venter and deep imprint zone.

Covariation and adaptive constraints are thus not mutually exclusive and both can contribute to parallel evolution of ammonoid lineages. Constructional constraints belong therefore to the primary factors governing evolutionary trends of the ammonoid shell, indirectly triggered by adaptive trends. Furthermore, this underlines that form, and the controls upon it, can never be truly understood in isolation from functional adaptation and constructional covariation. Distinction between covariation and adaptation in the process of evolutionary trends is also important in order to avoid over-interpretation of the patterns; in such cases, detailed studies of convergent or parallel evolutionary trends can contribute important impetus toward a decision for either cause. For instance, recurrence of particular combinations of morphology and their strong independence of phylogeny are commonly regarded as strong arguments for functional constraints. The evolutionary recurrence of these combinations of characters depends partially on selection for certain functional aspects (e.g., trend of increased involution induced by selection for improved hydrodynamic properties), and partially on shell morphogenesis and associated covariation following functional adaptations (e.g., trend of increased suture complexity induced by size- and involution-increase trends), thus representing "fabricational noise" [162,196] (i.e. constructional constraints). In other words, evolutionary transformations that occurred in these ammonoid lineages may be directly or indirectly linked to some kind of adaptation, but not all innovations are necessarily functional. For instance, some authors have interpreted the adaptive signification of sutural complexity, especially to water depth against implosion (for a discussion and references, see e.g., [144,190,197]). Such interpretations are questionable because trends in suture complexity may be (at least partially) a side effect and not the target of evolution. Furthermore, several studies also tried to demonstrate the increasing complexity of life by focusing on the ammonoid suture complexity (e.g., [36,198,199]). All these studies resulted in more or less equivocal results and this may be explained by the fact that many changes in suture patterns can be induced by covariation. It is thus crucial for phylogenetic analyses, especially at higher systematic ranks [200], to understand the driving factors behind evolutionary morphological modifications, whether they are driven by some selective force, sheer covariation or even random processes.

Those evolutionary trends which are not parallel between the two studied groups also highlights that these are independent characters of the ammonoid shell. Hence, although adaptation and covariation largely shape the morphological evolution of ammonoids, the still divergent evolution of several shell characters of both lineages in our case of parallel evolution imply that the unique histories of organisms still play a large role in shaping the evolutionary trajectory of clades [2]. As large-scale macroevolutionary studies can only proceed gradually, we hope that further fossil discoveries and the application of new methods and better knowledge of mollusc shell morphogenesis (see e.g., [164,165]) will help to test the hypotheses advocated in this paper, and continue to reveal information about the evolutionary history of this major marine extinct group, the ammonoids.

## Authors' contributions

CK designed the study. CK and KDB collected the data (field, museum, literature) and made the morphometric measurements. CM performed the quantitative analyses and edited the text. CM, CK and KDB wrote the paper. All authors participated in the interpretation of the data. All authors read, discussed and approved the final manuscript.
